# Association between Bilateral Selective Antegrade Cerebral Perfusion and Postoperative Ischemic Stroke in Patients with Emergency Surgery for Acute Type A Aortic Dissection—Single Centre Experience

**DOI:** 10.3390/medicina59081365

**Published:** 2023-07-26

**Authors:** Mircea Robu, Diana Romina Marian, Irina Margarint, Bogdan Radulescu, Ovidiu Știru, Andrei Iosifescu, Cristian Voica, Mihai Cacoveanu, Raluca Ciomag (Ianula), Bogdan Severus Gașpar, Lucian Dorobanțu, Vlad Anton Iliescu, Horațiu Moldovan

**Affiliations:** 1 Faculty of Medicine, Carol Davila University of Medicine and Pharmacy, 050474 Bucharest, Romania; 2 Prof. Dr. C.C. Iliescu Emergency Institute for Cardiovascular Diseases, 022322 Bucharest, Romania; 3 Department of Cardiovascular Surgery, Emergency Clinical Hospital Bucharest, 014461 Bucharest, Romania; 4 “Bagdasar-Arseni” Clinical Emergency Hospital, 041915 Bucharest, Romania; 5 Department of General Surgery, Bucharest Clinical Emergency Hospital, 014461 Bucharest, Romania; 6 Faculty of Medicine, Titu Maiorescu University, 040441 Bucharest, Romania; 7 Department of Cardiovascular Surgery, Monza Metropolitan Hospital, 040204 Bucharest, Romania; 8 Academy of Romanian Scientists, 54 Splaiul Independentei, 050711 Bucharest, Romania

**Keywords:** acute type A aortic dissection, stroke, antegrade cerebral perfusion, Hemiarch/aortic arch replacement

## Abstract

Acute type A aortic dissection (ATAAD) is a surgical emergency with a mortality of 1–2% per hour. Since its discovery over 200 years ago, surgical techniques for repairing a dissected aorta have evolved, and with the introduction of hypothermic circulatory arrest and cerebral perfusion, complex techniques for replacing the entire aortic arch were possible. However, postoperative neurological complications contribute significantly to mortality in this group of patients. The aim of this study was to determine the association between different bilateral selective antegrade cerebral perfusion (ACP) times and the incidence of postoperative ischemic stroke in patients with emergency surgery for ATAAD. Patients with documented hemorrhagic or ischemic stroke, clinical signs of stroke or neurological dysfunction prior to surgery, that died on the operating table or within 48 h after surgery, from whom the postoperative neurological status could not be assessed, and with incomplete medical records were excluded from this study. The diagnosis of postoperative stroke was made using head computed tomography imaging (CT) when clinical suspicion was raised by a neurologist in the immediate postoperative period. For selective bilateral antegrade cerebral perfusion, we used two balloon-tipped cannulas inserted under direct vision into the innominate artery and the left common carotid artery. Each cannula is connected to a separate pump with an independent pressure line. Near-infrared spectroscopy was used in all cases for cerebral oxygenation monitoring. The circulatory arrest was initiated after reaching a target core temperature of 25–28 °C. In total, 129 patients were included in this study. The incidence of postoperative ischemic stroke documented on a head CT was 24.8% (31 patients), and postoperative death was 20.9% (27 patients). The most common surgical technique performed was supravalvular ascending aorta and Hemiarch replacement with a Dacron graft in 69.8% (90 patients). The mean cardiopulmonary bypass time was 210 +/− 56.874 min, the mean aortic cross-clamp time was 114.775 +/− 34.602 min, and the mean cerebral perfusion time was 37.837 +/− 18.243 min. Using logistic regression, selective ACP of more than 40 min was independently associated with postoperative ischemic stroke (OR = 3.589; 95%CI = 1.418–9.085; *p* = 0.007). Considering the high incidence of postoperative stroke in our study population, we concluded that bilateral selective ACP should be used with caution, especially in patients with severely calcified ascending aorta and/or aortic arch and supra-aortic vessels. All efforts should be made to minimize the duration of circulatory arrest when using bilateral selective ACP with a target of less than 30 min, in hypothermia, at a body temperature of 25–28 °C.

## 1. Introduction

Acute type A aortic dissection (ATAAD) represents the most catastrophic pain syndrome since its discovery over 200 years ago due to high morbidity and mortality (1–2% per hour) [1,2,3]. Central aortic therapy with the main goal of resecting the primary intimal tear, stabilization of the aortic wall, end-organ protection, and restoring flow into the true lumen of the aorta is currently the preferred method of approach in ATAAD [4]. In 70% of cases, the aortic arch is dissected, or often the primary intimal tear extends or reenters within the aortic arch, and the supra-aortic vessels can be dissected or obstructed [5]. Hemiarch, complete arch replacement, or simply open distal anastomosis are performed during circulatory arrest and have an increased risk of neurological complications [1,6,7]. The incidence of neurologic complications after surgery for ATAAD is up to 32.8% in different studies [1,5,6,8]. Ischemic stroke after the repair of an ATAAD is associated with higher in-hospital mortality, longer intensive care and hospital stay, and reduced long-term survival [9,10]. While 6% of patients presented with ischemic stroke prior to emergency aortic repair, 12.7% of patients developed this complication after surgery [9]. Methods of cerebral protection have emerged to compensate for the brain’s low tolerance for ischemia. Retrograde cerebral perfusion (RCP) and antegrade cerebral perfusion (ACP) safely prolonged hypothermic circulatory arrest (HCA), one of the first strategies for brain protection. Studies showed that profound HCA at 10–15 °C for less than 30 min is safe [11,12]. ACP has more popularity than RCP, and it can be achieved either with a cannula in the right axillary artery after clamping the innominate artery (unilateral ACP) or by using two separate cannulas inserted directly into the innominate and left common carotid arteries (bilateral selective ACP). Unilateral ACP relies on the integrity of the circle of Willis [13,14], while bilateral selective ACP (BSACP) may increase the risk of stroke due to air embolism or dislodgement of atheromatous plaque when manipulating the arch vessels [15]. A meta-analysis conducted by Malvindi et al. showed that both techniques are acceptable, bilateral ACP being safer when HCA exceeds 40–50 min [14]. 

The aim of this study was to determine the association between different BSACP perfusion times and postoperative ischemic stroke in patients who underwent emergency surgery for ATAAD. 

## 2. Methods

Between January 2017 and December 2022, 220 patients were transferred to our center for ATAAD. The diagnosis was made based on chest computed tomography with intravenous contrast prior to arrival from tertiary centers. Demographic and clinical characteristics were collected from the medical records and electronic health system. 

Inclusion criteria: patients considered for emergent aortic repair for type A aortic dissection according to the Stanford classification on contrast chest CT with BSACP. 

We defined the following exclusion criteria: (1) patients with documented hemorrhagic or ischemic stroke based on CT scans prior to surgery; (2) clinical signs of a stroke or neurological dysfunction prior to surgery; (3) patients that died on the operating table or within 48 h after surgery; (4) patients in the postoperative period for whom the neurological status could not be assessed; (5) patients with incomplete preoperative, intraoperative or postoperative data. 

The diagnosis of ischemic stroke was made on head CT when clinical suspicion was raised in the immediate postoperative period (first 12 h) and after the neurological examination. The Modified Rankin Scale (mRS) was used to assess the degree of disability after ischemic stroke at discharge based on data collected from clinical charts and neurological examinations.

### 2.1. Surgical Technique 

**Figure 1:**
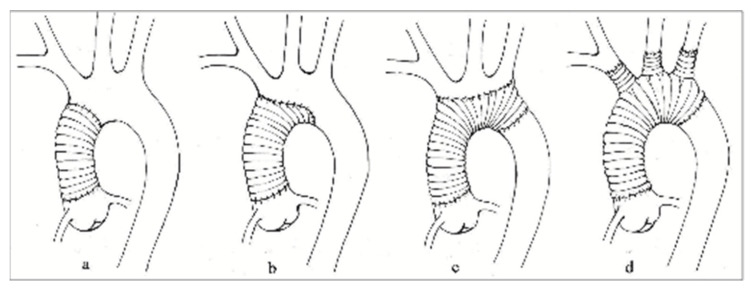
Technical options for ATAAD repair—(**a**) supracoronary ascending aorta replacement; (**b**) supracoronary ascending aorta and Hemiarch replacement; (**c**) supracoronary ascending aorta and total arch replacement, reconnection of supra-aortic arteries with the island technique; and (**d**) supracoronary ascending aorta and total arch replacement, separate reconnection of supra-aortic arteries.

After the institution of general anesthesia, a standard median sternotomy was performed. Institution of cardiopulmonary bypass (CPB) was conducted in most of the cases with a two-stage venous cannula in the right atrium and direct cannulation of axillary or femoral artery according to each case. Direct cannulation into the dissected ascending aorta was also performed if no other options were available. In cases that required concomitant mitral valve replacement, selective cannulation of the superior and inferior vena cava was conducted. The heart was arrested using cold (4 °C), crystalloid cardioplegia in anterior and retrograde fashion according to each case. Our institutional protocol consists of cooling the patient to a core temperature of 25–28 °C immediately after initiating CPB. Open distal anastomosis technique was performed with circulatory arrest and BSACP. A total arch replacement was performed only when the primary intimal tear could not be excluded with a Hemiarch approach. The reason behind this strategy was that complete arch replacement is associated with a greater risk of stroke than Hemiarch replacement [16,17]. Near-infrared spectroscopy (NIRS) was used in all cases to monitor cerebral oxygenation. BSACP protocol is as follows: when the target temperature is achieved and circulatory arrest initiated, the arch is opened, and two balloon-tipped cannulas are inserted under direct vision into the innominate artery and left common carotid artery. We use 13F or 15F cannulas according to the vessel’s diameter. Each cannula is connected to a different pump with a separate pressure monitoring line. For the left side, perfusion pressure is 60–70 mmHg with a flow of 180–230 mL/min. Right side perfusion pressure is 60–70 mmHg with a flow between 150 and 200 mL/min. Perfusion parameters are adapted within the mentioned intervals to reach a target value of 50–70% on NIRS. Before completing the distal anastomosis, the cannulas are extracted one at a time, and the innominate and left common carotid arteries are snared until completion of the anastomosis. After open distal anastomosis is performed, an arterial cannula is inserted directly into the Dacron prosthesis, CPB is restarted, and the supra-aortic vessels are unclamped after de-airing. During rewarming of the patient, the repair of ascending aorta and or aortic root is performed. Figure 1 shows the techniques most commonly used in our center. The rest of the operation is completed in the usual fashion. 

### 2.2. Statistical Analysis

Statistical analysis was conducted with Wizard 2 Statistical Software for Mac OS (Wizard–Statistics & Analysis^®^, Raipur, Chattisgarh, India). Summary statistics are presented as absolute numbers and percentages for categorical values and as the mean and standard deviation for continuous values. Our primary outcome was the development of new postoperative ischemic stroke in patients with emergency surgery for ATAAD with BSACP. We divided patients into three groups: BSACP < 30 min, between 30 and 40 min and >30 min. These thresholds were chosen considering that deep hypothermic circulatory arrest (DHCA) was safer under 30 min and ACP is safer when DHCA exceeds 40 min, as stated above [14]. In order to investigate the association between different BSACP perfusion times and ischemic stroke, multivariable analysis was performed using logistic regression and taking into account a model that included variables achieving a *p*-value < 0.1 in univariate analysis. A predictive modeling strategy with the backward stepwise method of entering data was then used. Variables included in the univariate analysis were the following: age, male sex, arterial hypertension, diabetes, dyslipidemia, preoperative atrial fibrillation, postoperative atrial fibrillation, cardiac tamponade at admission, severe aortic regurgitation, severe left ventricle dysfunction, presence of bicuspid aortic valve, severe calcifications of the ascending aorta or the aortic arch (as seen on preoperative chest CT), Hemiarch replacement, aortic root replacement, total arch replacement, supracoronary ascending aorta replacement, axillary cannulation, femoral cannulation, innominate artery dissection, left common carotid artery dissection, a primary intimal tear in the ascending aorta, and a primary entry tear in the aortic arch. Logistic regression results are presented as odds ratios (OR) with confidence limits and *p*-values. Sperman’s rank test was used to evaluate the correlation between different surgical techniques and postoperative ischemic stroke.

## 3. Results

After applying exclusion criteria, 91 patients were excluded, and 129 patients with emergency surgery for ATAAD with BSACP were included in this study. A total of 25 patients had documented ischemic or hemorrhagic stroke on head CT imaging, 5 patients arrived in coma (GCS 3), 12 patients had motor deficits, 11 patients died within 48 h after surgery, and 38 patients were excluded due to incomplete data. The preoperative data of the patients are summarized in Table 1. The mean age was 59 +/− 11.15 years, and 64.3% (83) were males. The mean Euroscore was 9.03 +/− 2.63. Arterial hypertension was the most common comorbidity found in more than half of the patients (65.9%), followed by dyslipidemia (31%). Severe calcifications in the ascending aorta or aortic arch were present in 7% of cases, according to preoperative chest CT. Data from preoperative transthoracic echocardiography showed severe aortic regurgitation in 18.6% of cases, the presence of a bicuspid aortic valve in 8.5% of cases, and cardiac tamponade in 27.1% of patients. The incidence of postoperative ischemic stroke was 24.8% (31 patients), and postoperative death was 20.9% (27 patients). The mean time from diagnosis to emergency surgery was 4.89 +/− 4.375 h. Causes of death based on autopsy are listed in Table 2, septic shock being the most frequent. Table 3 shows details about the surgical procedures and intraoperative data. The most frequently used technique for ATAAD was supracoronary ascending aorta and Hemiarch replacement with a Dacron graft in 69.8% of cases, followed by the ascending aorta and total arch replacement in 15.5% of cases and aortic root, ascending aorta and Hemiarch replacement in 10.9% of cases. A total of 13 patients required additional procedures (coronary artery bypass grafting with saphenous veins in 6 patients, mitral valve replacement in 4 patients, coarctation repair in one patient; one patient required peripheral veno-arterial extracorporeal membrane oxygenation (VA ECMO) after CPB and there was one case of femorofemoral bypass for leg ischemia following CPB. There was no correlation between the surgical technique and the development of new postoperative ischemic stroke (aortic arch replacement *p* = 0.732; supracoronary ascending aorta and Hemiarch replacement *p* = 0.678; aortic root replacement *p* = 0.650). The mean CPB time was 210 +/− 56.874 min, and the mean aortic cross-clamping time was 114.775 +/− 34.602 min. The mean cerebral perfusion time was 37.837 +/− 18.243 min. A total of 44.2% of the patients had less than 30 min of cerebral perfusion, 34.1% had cerebral perfusion of 30–40 min, and 31.8% had more than 40 min of cerebral perfusion. The most commonly used cannulation site was the axillary artery in 68.2% of patients, and 43.4% had the primary intimal tear in the ascending aorta. Table 4 shows the mRS of the patients with ischemic stroke at the moment of discharge from the hospital. The mean mRS was 2.611 +/− 1.037 in surviving patients.

### Logistic Regression

Results of the univariate analysis of variables achieving a *p*-value < 0.1 are presented in Table 5. Dissection of the left common carotid artery (OR = 2.772; 95%CI = 1.041–7.381; *p* = 0.041) and dyslipidemia (OR = 3.048; 95%CI =1.077–8.627; *p* = 0.036) were included in the final model after backward selection. Selective bilateral ACP over 40 min was associated with ischemic stroke (OR = 2.41; 95%CI = 1.054–5.509; *p* = 0.037) in univariate analysis and after model adjustment was an independent factor associated with postoperative ischemic stroke (OR = 3.589; 95%CI = 1.418–9.085; *p* = 0.007). Selective bilateral ACP time less than 30 min (OR = 0.484; 95%CI = 0.207–1.128; *p* = 0.093) and between 30 and 40 min (OR = 1.016; 95%CI = 0.438–2.357; *p* = 0.971) are not associated with ischemic stroke after univariate analysis (Table 6).

## 4. Discussion

ATAAD is a catastrophic event, and it is well established that without surgical treatment, mortality is 1–2% per hour. However, despite surgical advancements, the incidence of postoperative neurological complications is reported to be up to 32.8% in different studies [1,5,6,8]. In our study, the incidence of stroke was 21.6% (31 patients). A total of 13 (41.93%) of the patients with postoperative ischemic stroke died in the intensive care unit. The mean mRS in surviving patients was 2.611 +/− 1.037. In the group of surviving patients, four had a mRS of 4, and six had a mRS of 3, confirming the high mortality and morbidity of this postoperative complication. Open distal anastomosis, Hemiarch replacement, or complete aortic arch replacement require a period of circulatory arrest. Due to low brain tolerance to ischemia, methods for cerebral protection during this period have emerged. Historically DHCA (18–20 °C) was the first strategy used and is reported to be safe if used for under 30 min [11,12,18], with a significant increase in mortality if this threshold is exceeded [6,15]. Retrograde cerebral perfusion (RCP) via the superior vena cava and antegrade cerebral perfusion (ACP) via the right axillary artery or selective via cannulas inserted in the innominate artery and left common carotid artery have emerged for safely prolonging HCA. ACP is used in the majority of cases and has the advantage of superior flows to the brain in the normal flow direction [4]. Unilateral ACP relies on the integrity of the circle of Willis to perfuse the entire brain [13,16]. Some studies demonstrated the non-inferiority of unilateral ACP [13,17], and one study suggested that cerebral malperfusion is rare because of the reinforced flow to the brain via the right vertebral artery and extracranial collaterals [19]. Olsen et al. demonstrated that unilateral ACP has a risk of 6.6 greater than bilateral ACP for permanent neurologic dysfunction [20]. Bilateral selective ACP may increase the risk of stroke due to air embolism or dislodgement of atheromatous plaques when manipulating the arch vessels [15]. The institution of bilateral selective ACP is more technically complex, and studies demonstrated longer total circulatory arrest time with this technique with an increased risk of stroke if it exceeds 30 min [19,21].

In our center, we use BSACP in all patients requiring HCA for aortic repair in ATAAD. The technique is adapted based on intraoperative findings, especially if atheromatous plaques are present in the aortic arch or at the level of the innominate artery and left common carotid artery. If supra-aortic vessels are implanted separately, we usually excise the portion with an atheromatous plaque for safe insertion of the cannulas. Also, in these cases, no snaring is conducted in order to minimize the risk of plaque embolization. Also, the portion dissected of the supra-aortic vessels is excised as much as possible in order to obtain an optimal pressure for cerebral perfusion. Cannulas are inserted after de-airing, and we try to insert them as minimally as possible in the arterial lumen, usually with the balloon adjacent to the proximal end of the artery. The circuits for ACP were prepared prior to HCA in order to minimize time loss, and separate color codes were assigned for the left and right cannulas in order to better manage cerebral perfusion. Using this algorithm, the mean cerebral perfusion time in our study was 37,837 +/− 18,243 min, and we managed to have an ACP of less than 30 min in 44.2% of patients. Out of 90 patients with Hemiarch replacement, 75.4% had BSACP less than 30 min, and 90.5% with a mean aortic cross-clamp between 85 and 96 min. Regarding that BSACP is technically more complex, based on our results, we do not think this is the case. The incidence of postoperative ischemic stroke was 24.8% in our study. Out of 88 patients with BSACP > 40 min, ischemic stroke occurred in more than half (53.1%). We concluded that every effort should be made to minimize ACP time.

There is a continuous debate regarding optimal pressure and flow during ACP. Studies using animal models reported cerebral edema and inferior neurobehavioral recovery when perfusion pressure was high (90 mmHg), although cerebral flow increased with higher pressures [22]. Spielvogel and colleagues proposed, after a review of all strategies, the following protocol for selective ACP: perfusion pressure between 40 and 60 mmHg, flow rates from 6 to 10 mL/kg/min, and core cooling between 18 and 30 °C [23].

Our institutional BSACP protocol is as follows: after the target core temperature is achieved (26–28 °C) and circulatory arrest initiated, the arch is opened, and two balloon-tipped cannulas are inserted under direct vision into the innominate artery and left common carotid artery. We use 13F or 15F cannulas according to the vessel’s diameter. Each cannula is connected to a different pump and has a separate pressure monitoring line. For the left side, perfusion pressure is 60–70 mmHg with a flow of 180–230 mL/min. Right side perfusion pressure is 60–70 mmHg with a flow between 150 and 200 mL/min. Perfusion parameters are adapted within the mentioned parameters to reach a 50–70% value on NIRS. We consider NIRS a valuable tool for managing cerebral flow during surgery for ATAAD. Low values of NIRS in the moment of aortic cross-clamping mandate changing the cannulation site.

Recent studies found a lower incidence of ischemic stroke after surgery for ATAAD than our result [10,24]. Dumfarth et al. found a 15.8% incidence of stroke in these patients, significantly lower than our results. They used both antegrade and retrograde cerebral perfusion with a mean HCA time of 44.9 +/− 21.3 min. Patients with stroke had longer CPB time (stroke: 283.8 ± 133 min vs. no stroke: 235 ± 82 min, *p* = 0.017) and less common axillary artery cannulation (stroke: 31.3% vs. no stroke: 47.6%, *p* = 0.040). We think that our higher incidence of ischemic stroke can be due to a longer period of time from diagnosis to surgery (4.89 +/− 4.375 h), considering the 1–2% mortality per hour in this group of patients. Also, using exclusive BSACP can explain our higher incidence of ischemic stroke when considering the risk of air or atheromatous plaque embolism in the moment of cannulas insertion or manipulation during distal anastomosis. Regarding the site of cannulation, 75% of patients with ischemic stroke in our study had femoral artery cannulation, and 36.4% had CPB time longer than 280 min supporting the data presented by Dumfarth et al. Hongliang et al. found an incidence of 18.4% of cerebral infarction post-surgery for ATAAD, and they concluded that particular attention should be given to false lumen thrombosis, aortic arch entry, and coronary artery involvement to avoid postoperative stroke. While we do not have sufficient data to investigate coronary artery involvement and false lumen thrombosis, we found that 70.4% of patients had aortic arch entry in accordance with the above results.

Further studies are needed to establish the optimal protocol to minimize cerebral ischemia.

## 5. Conclusions

Considering the high incidence of postoperative stroke in our study population (24.8%), we concluded that bilateral selective ACP should be used with caution, especially in patients with severely calcified ascending aorta and or aortic arch and supra-aortic vessels. Careful manipulation of these vessels when introducing and securing the balloon tips cannulas and de-airing of the vessels at the conclusion of distal anastomosis is mandatory. Perfusion parameters should be carefully adapted in each patient according to NIRS values. All efforts should be conducted to minimize the duration of circulatory arrest when using bilateral selective ACP with a target of less than 30 min at a body temperature of 25–28 °C.

## Figures and Tables

**Table 1:** Preoperative characteristics.

Parameter (Unit)	*n* = 129 (100%)
Age (mean, SD)	59 (11.15)
Gender (*n*, % male)	83 (64.3%)
Euroscore (mean, SD)	9.03 (2.63)
Time from diagnostic CT to surgery (hours)	4.89 (4.375)
Arterial Hypertension (*n*, %)	85 (65.9%)
Diabetes (*n*, %)	8 (6.2%)
Dyslipidemia (*n*, %)	40 (31%)
Chronic Kidney Disease (*n*, %)	10 (7.8%)
Preoperative Atrial Fibrillation (*n*, %)	12 (9.3%)
Bicuspid Aortic Valve (*n*, %)	11 (8.5%)
Cardiac Tamponade at Admission (*n*, %)	35 (27.1%)
Dissection of Innominate Artery (*n*, %)	33 (25.6%)
Dissection of Left Common Carotid Artery (*n*, %)	21 (16.3%)
Dissection of Innominate Artery and Left Common Carotid Artery (*n*, %)	9 (6.97%)
Severe Aortic Regurgitation (*n*, %)	24 (18.6%)
Mild left ventricular dysfunction (LVEF 40–50%) (*n*, %)	9 (7%)
Moderate left ventricle dysfunction (LVEF 30–40%) (*n*, %)	2 (1.6%)
Severe left ventricle dysfunction (LVEF < 30%) (*n*, %)	1 (0.8%)
Severe calcifications of ascending aorta or aortic arch (*n*, %)	9 (7%)

LVEF: left ventricle ejection fraction.

**Table 2:** Causes of death based on autopsy results.

Death Cause	No (%)
Cardiogenic shock	7 (5.4)
Septic shock	12 (9.3)
Hemorrhagic stroke	2 (1.6)
Mixed shock (cardiogenic and septic)	6 (4.7)

**Table 3:** Intraoperative data.

Parameter (Unit)	*n* = 129 (100%)
Type of operation	
Supracoronary ascending aorta and Hemiarch replacement (*n*, %)	90 (69.8%)
Supracoronary ascending aorta and arch replacement (*n*, %)	20 (15.5%)
Aortic root, ascending aorta, and Hemiarch replacement (*n*, %)	14 (10.9%)
Supracoronary ascending aorta replacement (*n*, %)	3 (2.3%)
Aortic root, ascending aorta, and arch replacement (*n*, %)	1 (0.8%)
Combined procedures	13 (10.07%)
Mitral valve replacement (*n*, %)	4 (3.1%)
Coronary artery bypass grafting (*n*, %)	6 (4.65%)
Peripheral V-A ECMO (*n*, %)	1 (0,8%)
Femorofemoral Bypass (*n*, %)	1 (0.8%)
Aortic coarctation repair (*n*, %)	1 (0.8%)
Cannulation site	
Axillary artery (*n*, %)	88 (68.2%)
Femoral artery (*n*, %)	39 (30.2%)
Aortic arch (*n*, %)	2 (1.6%)
Primary entry tear	
Ascending aorta/aortic root (*n*, %)	56 (43.4%)
Aortic arch (*n*, %)	42 (32.6%)
Ascending aorta/aortic root and aortic arch (*n*, %)	12 (9.3%)
Not found in the aortic arch or ascending aorta/aortic root (*n*, %)	18 (14%)
Cardiopulmonary bypass time (min); (mean, SD)	210 (56,874)
Aortic cross-clamp time (min); (mean, SD)	114,775 (34,602)
Cerebral perfusion time (min); (mean, SD)	37,837 (18,243)
Cerebral perfusion below 30 min (*n*, %)	57 (44.2)
Cerebral perfusion between 30 and 40 min (*n*, %)	44 (34.1)
Cerebral perfusion over 40 min (*n*, %)	41 (31.8)
Postoperative atrial fibrillation (*n*, %)	34 (26.4)
Subsequent intervention for bleeding (*n*, %)	34 (26.4)

**Table 4:** Modified Rankin Scale (mRS) at discharge.

mRS	*n*/(100%)
0	0 (0%)
1	3 (9.67%)
2	5 (16.12%)
3	6 (19.35%)
4	4 (12.90%)
5	0 (0%)
6	13 (41.93%)

**Table 5:** Factors associated with ischemic stroke (multivariable statistics).

	Univariate Analysis			Multivariable Analysis		
	OR	95%CI	*p*	OR	95%CI	*p*
LCCAD	2.772	1.041–7.381	0.041	2.941	1.034–8.364	0.043
Dyslipidemia	3.048	1.077–8.627	0.036	4.577	1.462–14.332	0.009
BSACP > 40	2.41	1.054–5.509	0.037	3.589	1.418–9.085	0.007

LCCAD: left common carotid artery dissection; BSACP > 40: selective bilateral antegrade cerebral perfusion more than 40 min.

**Table 6:** Single factor analysis of selective bilateral ACP time under 30 min and between 30 and 40 min.

	OR	95%CI	*p*
BSACP 30–40 min	1.016	0.438–2.357	0.971
BSACP < 30 min	0.484	0.207–1.128	0.093

BSACP: bilateral selective antegrade cerebral perfusion.

## Data Availability

The data presented in this study are available on reasonable request from the corresponding author.

## References

[1] Tsai T.T., Trimarchi S., Nienaber C.A. (2009). Acute aortic dissection: Perspectives from the International Registry of Acute Aortic Dissection (IRAD). Eur. J. Vasc. Endovasc. Surg..

[2] Costache V.S., Meekel J.P., Costache A., Melnic T., Solomon C., Chitic A.M., Bucurenciu C., Moldovan H., Antoniac I., Candea G. (2020). Geometric analysis of type B aortic dissections shows aortic remodeling after intervention using multilayer stents. Materials.

[3] Robu M., Marian D.R., Vasile R., Radulescu B., Stegaru A., Voica C., Nica C., Gheorghita D., Zaharia O., Iulian A. (2022). Delayed Surgical Management of Acute Type A Aortic Dissection in a Patient with Recent COVID-19 Infection and Post-COVID-19 Bronchopneumonia—Case Report and Review of Literature. Medicina.

[4] Gemelli M., Di Tommaso E., Natali R., Dixon L.K., Mohamed Ahmed E., Rajakaruna C., Bruno V.D. (2023). Validation of the German Registry for Acute Aortic Dissection Type A Score in predicting 30-day mortality after type A aortic dissection surgery. Eur. J. Cardiothorac. Surg..

[5] Ehrlich M.P., Ergin M.A., McCullough J.N., Lansman S.L., Galla J.D., Bodian C.A., Apaydin A., Griepp R.B. (2000). Results of immediate surgical treatment of all acute type A dissections. Circulation.

[6] Easo J., Weigang E., Hölzl P.P., Horst M., Hoffmann I., Blettner M., Dapunt O.E. (2013). Influence of operative strategy for the aortic arch in DeBakey type I aortic dissection—Analysis of the German Registry for Acute Aortic Dissection type A (GERAADA). Ann. Cardiothorac. Surg..

[7] Moldovan H., Bulescu C., Sibisan A.M., Tiganasu R., Cacoveanu C., Nica C., Rachieru A., Gheorghita D., Zaharia O., Balanescu S. (2021). A Large Ascending Aorta Thrombus in a Patient with Acute Myocardial Infarction—Case Report. Medicina.

[8] Conzelmann L.O., Hoffmann I., Blettner M., Kallenbach K., Karck M., Dapunt O., Borger M.A., Weigang E., GERAADA Investigators (2012). Analysis of risk factors for neurological dysfunction in patients with acute aortic dissection type A: Data from the German Registry for Acute Aortic Dissection type A (GERAADA). Eur. J. Cardiothorac. Surg..

[9] Ghoreishi M., Sundt T.M., Cameron D.E., Holmes S.D., Roselli E.E., Pasrija C., Gammie J.S., Patel H.J., Bavaria J.E., Svensson L.G. (2020). Factors associated with acute stroke after type A aortic dissection repair: An analysis of the Society of Thoracic Surgeons National Adult Cardiac Surgery Database. J. Thorac. Cardiovasc. Surg..

[10] Dumfarth J., Kofler M., Stastny L., Plaikner M., Krapf C., Semsroth S., Grimm M. (2018). Stroke after emergent surgery for acute type A aortic dissection: Predictors, outcome and neurological recovery. Eur. J. Cardiothorac. Surg..

[11] Svensson L.G., Crawford E.S., Hess K.R., Coselli J.S., Raskin S., Shenaq S.A., Safi H.J. (1993). Deep hypothermia with circulatory arrest. Determinants of stroke and early mortality in 656 patients. J. Thorac. Cardiovasc. Surg..

[12] Dong S.B., Xiong J.X., Zhang K., Zheng J., Xu S.D., Liu Y.M., Sun L.Z., Pan X.D. (2020). Different hypothermic and cerebral perfusion strategies in extended arch replacement for acute type a aortic dissection: A retrospective comparative study. J. Cardiothorac. Surg..

[13] Liu Z., Wang C., Zhang X., Wu S., Fang C., Pang X. (2020). Effect of different types of cerebral perfusion for acute type A aortic dissection undergoing aortic arch procedure, unilateral versus bilateral. BMC Surg..

[14] Malvindi P.G., Scrascia G., Vitale N. (2008). Is unilateral antegrade cerebral perfusion equivalent to bilateral cerebral perfusion for patients undergoing aortic arch surgery?. Interact. Cardiovasc. Thorac. Surg..

[15] Kazui T., Inoue N., Komatsu S. (1989). Surgical treatment of aneurysms of the transverse aortic arch. J. Cardiovasc. Surg..

[16] Immer F.F., Moser B., Krähenbühl E.S., Englberger L., Stalder M., Eckstein F.S., Carrel T. (2008). Arterial access through the right subclavian artery in surgery of the aortic arch improves neurologic outcome and mid-term quality of life. Ann. Thorac. Surg..

[17] Zierer A., El-Sayed Ahmad A., Papadopoulos N., Moritz A., Diegeler A., Urbanski P.P. (2012). Selective antegrade cerebral perfusion and mild (28 °C–30 °C) systemic hypothermic circulatory arrest for aortic arch replacement: Results from 1002 patients. J. Thorac. Cardiovasc. Surg..

[18] Ergin M.A., Galla J.D., Lansman S.L., Quintana C., Bodian C., Griepp R.B. (1994). Hypothermic circulatory arrest in operations on the thoracic aorta. Determinants of operative mortality and neurologic outcome. J. Thorac. Cardiovasc. Surg..

[19] Song S.J., Kim W.K., Kim T.H., Song S.W. (2022). Unilateral versus bilateral antegrade cerebral perfusion during surgical repair for patients with acute type A aortic dissection. JTCVS Open.

[20] Olsson C., Thelin S. (2006). Antegrade cerebral perfusion with a simplified technique: Unilateral versus bilateral perfusion. Ann. Thorac. Surg..

[21] Preventza O., Simpson K.H., Cooley D.A., Cornwell L., Bakaeen F.G., Omer S., Rodriguez V., de la Cruz K.I., Rosengart T., Coselli J.S. (2015). Unilateral versus bilateral cerebral perfusion for acute type A aortic dissection. Ann. Thorac. Surg..

[22] Halstead J.C., Meier M., Wurm M., Zhang N., Spielvogel D., Weisz D., Bodian C., Griepp R.B. (2008). Optimizing selective cerebral perfusion: Deleterious effects of high perfusion pressures. J. Thorac. Cardiovasc. Surg..

[23] Spielvogel D., Tang G.H. (2013). Selective cerebral perfusion for cerebral protection: What we do know. Ann. Cardiothorac. Surg..

[24] Zhao H., Guo F., Xu J., Zhu Y., Wen D., Duan W., Zheng M. (2020). Preoperative Imaging Risk Findings for Postoperative New Stroke in Patients With Acute Type A Aortic Dissection. Front. Cardiovasc. Med..

